# Using the contribution matrix to evaluate complex study limitations in a network meta-analysis: a case study of bipolar maintenance pharmacotherapy review

**DOI:** 10.1186/s13104-016-2019-1

**Published:** 2016-04-14

**Authors:** Toshi A. Furukawa, Tomofumi Miura, Anna Chaimani, Stefan Leucht, Andrea Cipriani, Hisashi Noma, Hiroshi Mitsuyasu, Shegenobu Kanba, Georgia Salanti

**Affiliations:** Department of Health Promotion and Human Behavior, Kyoto University Graduate School of Medicine/School of Public Health, Yoshida Konoe-cho, Sakyo-ku, Kyoto, 606-8501 Japan; Department of Neuropsychiatry Graduate School of Medical Sciences, Kyushu University, 3-1-1 Maidashi, Higashi-ku, Fukuoka, 812-8582 Japan; Department of Hygiene and Epidemiology, University of Ioannina School of Medicine, 45110 Ioannina, Greece; Department of Psychiatry and Psychotherapy, Technische Universität München, Ismaningerstr. 22, 81675 Munich, Germany; Department of Psychiatry, University of Oxford, Warneford Lane, Headington, Oxford, OX3 7JX UK; Department of Data Science, The Institute of Statistical Mathematics, 10-3 Midori-cho, Tachikawa, Tokyo, 190-8562 Japan; Institute of Social and Preventive Medicine (ISPM) & Berner Institut für Hausarztmedizin (BIHAM), University of Bern, Finkenhubelweg 11, 3012 Bern, Switzerland

**Keywords:** Network meta-analysis, GRADE, Study limitations, Risk of bias, Enrichment design

## Abstract

**Background:**

Limitations in the primary studies constitute one important factor to be considered in the grading of recommendations assessment, development, and evaluation (GRADE) system of rating quality of evidence. However, in the network meta-analysis (NMA), such evaluation poses a special challenge because each network estimate receives different amounts of contributions from various studies via direct as well as indirect routes and because some biases have directions whose repercussion in the network can be complicated.

**Findings:**

In this report we use the NMA of maintenance pharmacotherapy of bipolar disorder (17 interventions, 33 studies) and demonstrate how to quantitatively evaluate the impact of study limitations using *netweight*, a STATA command for NMA. For each network estimate, the percentage of contributions from direct comparisons at high, moderate or low risk of bias were quantified, respectively. This method has proven flexible enough to accommodate complex biases with direction, such as the one due to the enrichment design seen in some trials of bipolar maintenance pharmacotherapy.

**Conclusions:**

Using *netweight*, therefore, we can evaluate in a transparent and quantitative manner how study limitations of individual studies in the NMA impact on the quality of evidence of each network estimate, even when such limitations have clear directions.

## Background

The number of network meta-analyses (NMA) has been increasing rapidly in recent years [1], and concomitantly the methodology for NMA is also quickly developing and expanding. One of the most important topics around NMA currently is how we should assess the quality of evidence provided by NMA. Two papers have been published recently that attempt to apply the grading of recommendations assessment, development, and evaluation (GRADE) system of rating quality of evidence to NMA [2, 3].

According to GRADE, various components impact on the quality of findings from systematic reviews. Limitations in the primary studies constitute one important factor that can influence the quality of the pooled estimates. In traditional pairwise meta-analyses, the evaluation of study limitations of the included studies is fairly straightforward because one can visualise each study’s risks of bias in a table format and then evaluate their contributions to the pairwise meta-analytic results directly. On the other hand, NMA poses a special challenge in this assessment because different NMA estimates receive different amounts of contributions from all the studies in the network via direct as well as indirect contributions, and their respective contributions are not apparent.

The method proposed by Puhan et al. [2] rates the quality of evidence separately for direct and indirect estimates, and each rating is more impressionistic than quantitative. Moreover, when the network has many nodes and is more complex than triangular, they recommend focusing on the so-called first order loop (i.e. the triangular loop) for examination of the indirect estimates and suggests using the higher of the two ratings as the rating of the network estimate. In other words this method fails to take into account the remaining contributions. The authors therefore calls for research in how to use weights of individual studies in evaluating quality of NMA estimates [2]. The method proposed by Salanti et al. [3] uses weights more extensively and makes more quantitative evaluations of all the involved evidence. We applied this method in a previous NMA on maintenance pharmacotherapy of bipolar disorder [4], while paying due attention to the amount of contribution from each individual study.

### The problem of “enrichment design” in bipolar maintenance pharmacotherapy studies

The appraisal of the impact of study limitations in the NMA of the maintenance pharmacotherapy of bipolar disorder presents an additional interesting feature that renders this assessment even more challenging.

Bipolar disorder is a psychiatric disease in which patients typically show recurrent episodes of both manic and depressive episodes. While acute treatment is aimed at treating the acutely manic or depressive symptoms, long-term maintenance treatment is usually necessary to minimise the risk of recurrence of both manic and depressive episodes. Bipolar patients recruited into maintenance or prophylactic studies are usually in an euthymic phase, without acute symptoms. In some of these clinical trials, however, only the participants who had achieved remission of the index acute manic or depressive episode by treatment with a certain drug were included in the maintenance phase of the trial and then were randomised to continue the same drug or switch to another active drug (or placebo). Such a study design is called ‘enrichment design’, as it is ‘enriched’ by patients whose acute manic or depressive episode had responded to the drug used in the acute phase.

This study design has many limitations [5]. In particular, its results will tend to favour the drug that was effective in the acute phase mainly in the prevention of future episodes of the same polarity as the index episode and not necessarily in the prevention of episodes of the opposite polarity. The risk of bias due to the enrichment design therefore has a direction. For example, if a study included only those who had remitted from a manic episode on drug X and randomised them to continue on drug X or to switch to drug Y in order to compare these interventions’ efficacy in preventing a new manic or depressive episode, it is easy to foresee that such patients’ future manic episodes would be relatively responsive to drug X but possibly not their depressive episodes. On the other hand, drug Y is clearly not favoured in any direction as the patients had been originally selected as responders to drug X.

In the present article we use a published NMA as a working example and present a transparent and systematic method to assess how study limitations of individual randomised controlled trials (RCTs), including those due to the enrichment design, impact on the quality of evidence in the NMA. In NMA, it is almost certain that confidence in estimates will vary from comparison to comparison. We therefore essayed to appraise the quality of evidence for each comparison contained in the network. In the following we will illustrate how study limitations without direction (i.e. risks of bias usually assessed according to the Cochrane Handbook) and then those with direction (i.e. risk of bias due to the enrichment design) can be quantitatively summarised and evaluated to characterise each network estimate.

## Methods

### Materials

**Fig. 1:**
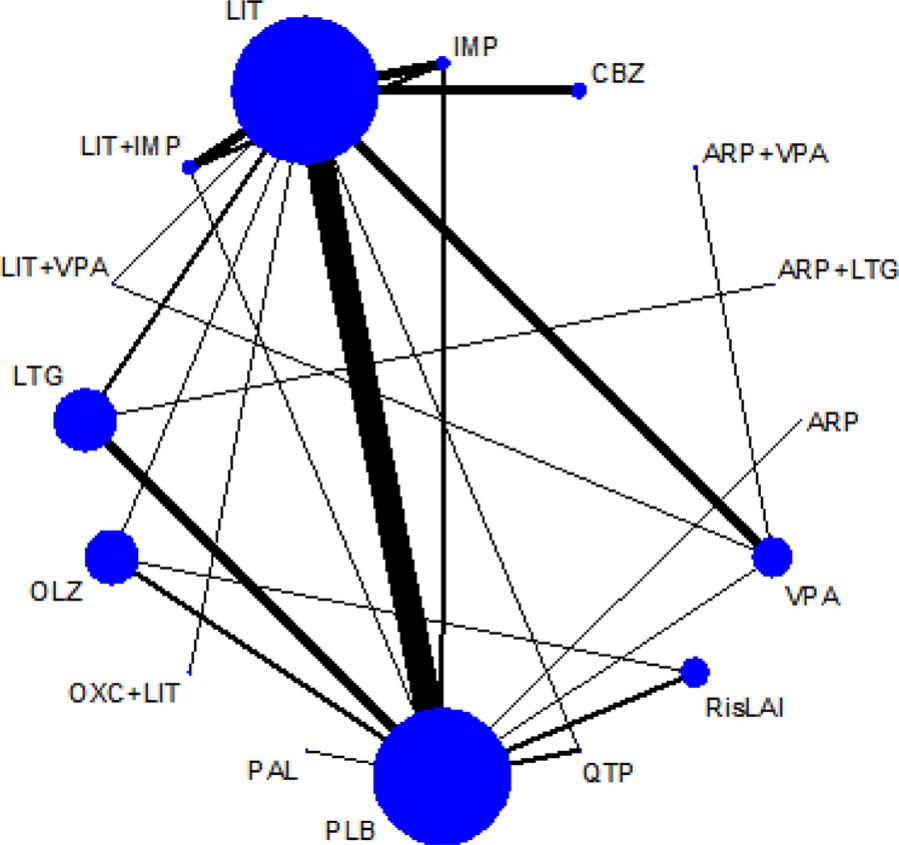
Network of eligible comparisons in the multiple-treatment meta-analysis for any mood episode relapse. Each node (*circle*) corresponds to a drug included in the analyses, with the size proportional to the number of participants assigned to that drug. Each *line* represents different comparisons between drugs, with the width of the line proportional to the number of trials comparing each pair of treatments. *ARP* aripiprazole, *CBZ* carbamazepine, *FLX* fluoxetine, *IMP* imipramine, *LIT* lithium, *LTG* lamotrigine, *OLZ* olanzapine, *OXC* oxcarbazepine, *PAL* paliperidone, *PLB* placebo, *QTP* quetiapine, *RisLAI* risperidone long-acting injection, *VPA* valproate

The NMA in question represents a systematic review of randomised controlled trials that compared active treatments for bipolar disorder (or placebo), either as monotherapy or as add-on treatment, for at least 12 weeks [4]. The primary outcome was the number of participants with recurrence of any mood episode this primary outcome was a combination of two secondary outcomes, namely the number of participants with recurrence of a manic episode and those with recurrence of a depressive episode. All in all we identified and included 33 randomised controlled trials that examined 17 maintenance pharmacotherapies for bipolar disorder in 6846 participants. Figure 1 shows the network formed by the identified comparisons in this NMA. We conducted a random-effects network meta-analysis within a Bayesian framework using Markov chain Monte Carlo in OpenBUGS 3.2.2. [6].

### Assessment of risk of bias of each study and of each direct comparison

Two assessors rated the risk of bias (RoB) of each RCT according to the Cochrane Handbook risk of bias tool [7]. The RoB examines the key methodological issues in a randomised trial, such as generation of random sequence, concealment of allocation, blinding of participants, blinding of therapists, blinding of outcome assessment, incomplete outcome data, and selective outcome reporting. We also assessed whether the definitions of the mood episode relapse or recurrence were explicit/operationalised or not in the primary studies, and the sponsorship bias. We rated an item at unclear risk of bias when we did not find sufficient information to judge it at either high or low risk.

Then we made a summary evaluation of RoB for each included study according to the following categories:

- Low risk of bias: there is no item rated at high risk among the nine items listed above.
- Moderate risk of bias: there is one item rated at high risk.
- High risk of bias: there are two or more items rated at high risk.

We examined the validity of this classification by pooling and comparing RR for studies rated as low, moderate or high risk of bias in a comparison if this comparison had an enough number of included trials to enable such validation.

After making a summary evaluation of RoB for each study, we made a similar evaluation of RoB for each direct comparison. When studies rated at different risks of bias were pooled, we made a summary evaluation by taking into account the weight that each study is given in pooling the studies into one direct comparison estimate as follows:

- Low risk of bias: all the included studies were rated as low risk of bias.
- Moderate risk of bias: all the studies were rated as moderate or low risk of bias; or there was one study rated as high risk of bias but this study contributed less than one quarter of the pooled sample.
- High risk of bias: there are two or more studies rated at high risk; or one major study at high risk of bias made a substantial contribution.

The above method of summarising RoBs of various domains into RoB of a study and then summarising study RoBs into RoB of a comparison is admittedly to a certain extent arbitrary. However, it must be noted that we can use the same logic and calculations, as we demonstrate below, to synthesise these characteristics at the level of each pairwise comparison into those at the level of each network estimate. In the following we shall therefore use the definitions above to illustrate our method.

### Assessment of ‘enrichment design’ for each study and for each direct comparison

We also evaluated whether each study used the enrichment design in relation with the polarity of the mood episode. The influence of the enrichment design was assessed separately for the two secondary outcomes: prevention of depressive episodes and prevention of manic episodes. Participants were considered to be enriched for a certain drug for depressive episode relapse (*depressive enrichment*) when they had been recruited at an acute depressive episode and investigated for the depressive episode relapse after being stabilised by that drug, and participants were considered to be enriched for a drug for manic episode relapse (*manic enrichment*) when they had been recruited at an acute manic episode and investigated for the manic episode relapse after being stabilized by that drug.

We first calculated the percentages of both depressive and manic enrichment for each study according to the number of participants in acute depressive or manic episode at recruitment, and then we estimated the corresponding percentages for each direct comparison consisting of one or more studies with consideration of the direction of enrichment for each study. For example, if a direct comparison A vs B consisted of two studies, one of which (n = 100) did not use the enrichment design but the other (n = 200) recruited patients at their depressive episodes and treated them with drug A, then this direct comparison would have 67 % (200/300) of participants enriched for depressive relapse in favour of drug A, 33 % not enriched for depressive relapse and 100 % not enriched for manic relapse.

### Using the contribution matrix to quantify the influence of RoB and of enrichment design in each network estimate

We used a recently developed tool for NMA, called the contribution matrix, that quantifies how much each direct comparison in the network contributes to each network estimate in the NMA [8, 9].

Let’s take a simple, triangular network ABC. We first calculate the direct estimate comparing A vs B, A vs C and B vs C by pooling trials comparing A vs B, A vs C, and B vs C, respectively. We denote these as D_AB_, D_AC_ and D_BC_. In the NMA of the full triangle, the mixed or network estimate comparing A vs B comes from the direct comparison D_AB_ and the indirect comparison I_AB_ consisting of D_BC_ and D_CA_ via C. For the simple situation in which each of the direct estimates has the same variance, the network estimate N_AB_ is (2*D_AB_ + (D_AC−_D_BC_))/3. Thus, for the mixed estimate (or also called network estimate) N_AB_, the three direct estimates D_AB_, D_AC_ and D_BC_ makes contributions of 50, 25 and 25 %, respectively.

When the network structure is complex and when variances are not equal, calculating the contribution of each direct estimate to each network estimate in the NMA is more complicated. In general more weight is given to direct comparisons with more precision and to those that are more central to the network and thus contribute to more indirect comparisons. Using the *netweight* command in Stata [10], we calculated the contribution matrix showing contributions from each direct comparison to the network comparisons. The weight that each direct comparison contributes to the network estimates is a combination of the variance of the direct comparison and the network structure: a comparison with much direct information not only contributes much to the network estimate of that comparison but also is more influential on its neighboring comparisons than its remotely placed comparisons, and a comparison for which little direct evidence exists benefits most from the rest of the network. Using *netweight,*^1^ the percentage contribution of each direct comparison to each network estimate is summarised in a matrix with rows representing network estimates and columns representing the available direct comparison in the network.

In order to characterize the RoB of each network estimate, we multiplied the contributions from direct comparisons at low, moderate or high risk of bias, respectively, by the contribution percentage that each direct estimate is making to the network estimate. This calculation provided the percentage of contributions from direct estimates rated at low, moderate or high risk of bias, respectively, to each network estimate.

In order to quantify the contribution from enrichment design to each network estimate, we multiplied the percentage of enrichment for each direct comparison by the contribution percentage that each direct estimate is making to the network estimate. For a particular network estimate of A vs B, this calculation provided the percentage of contributions from enriched studies favouring A, those favouring B, those dis-favouring A (i.e. favouring another drug C over A), those dis-favouring B, and those that involve neither A nor B (enrichment of unknown direction). The remaining came from non-enriched studies. We summed up the percentage of contributions from studies favouring A and those dis-favouring B as the percentage of enrichment favouring A. In the same manner, the percentage of enrichment favouring B was calculated by summing up the percentage of contributions from studies favouring B and those dis-favouring A.

## Results

### RoB of network estimates

Table 1 lists RoB for each individual study, and the summary assessment of RoB for each direct comparison, following the general rules as described in the methods. Placebo vs lithium was the only comparison where we had an enough number of trials at high, moderate or low risks of bias to compare the effect estimates for the same underlying true effect. Pooled estimates of lithium over placebo in prevention of any mood episode for studies assesses as being at high, moderate and low risks of bias were 0.58 (95 % CI 0.47–0.71), 0.60 (0.52–0.69) and 0.80 (0.54–1.19) in the theoretically expected ascending order, thus validating our assessment of RoB.

**Table 1:** Risk of bias assessments for each individual study and for each direct comparison against placebo

Comparison	Study	N	Risks of bias									RoB of each study and of comparison
			Sequence generation	Allocation concealment	Blinding of participant	Blinding of therapist	Blinding of assessor	Incomplete outcome data	Selective reporting	Definition of recurrent mood episode	Sponsorship	
PLB vs LIT	Melia 1970	11	U	U	L	L	L	U	L	H	H	H
	Cundall 1972	13	U	U	L	L	L	L	U	H	U	M
	Prien 1973a	31	U	U	L	H	L	L	H	H	L	H
	Prien 1973b	205	U	U	L	H	L	L	H	H	L	H
	Dunner 1976	40	U	U	L	L	L	L	H	H	U	H
	Fieve 1976	53	U	U	L	L	L	L	L	H	L	M
	Bowden 2000	185	U	U	L	L	L	L	L	L	L	L
	Bowden 2003	116	U	U	L	L	L	L	L	H	L	M
	Calabrese 2003	242	U	U	L	L	L	L	L	H	L	M
	Amsterdam 2010	53	U	U	L	L	L	L	L	L	L	L
	Weisler 2011	764	L	L	L	L	L	H	L	L	L	M
PLB vs VPA	Pooled											H
	Bowden 2000	281	U	U	L	L	L	L	L	L	H	M
PLB vs LTG	Pooled											M
	Calabrese 2000	182	U	U	L	L	L	L	L	H	H	H
	Bowden 2003	129	U	U	L	L	L	L	L	H	H	H
	Calabrese 2003	292	U	U	L	L	L	L	L	H	H	H
	Koyama 2011	103	U	U	L	L	L	L	L	H	H	H
PLB vs IMP	Pooled											H
	Prien 1973a	26	U	U	L	H	L	L	H	H	L	H
	Kane 1982	12	U	U	L	L	L	L	L	L	L	L
PLB vs LIT + IMP	Pooled											H
	Kane 1982	13	U	U	L	L	L	L	L	L	L	L
PLB vs ARP	Pooled											L
	Keck 2007	161	U	U	L	L	L	L	L	L	H	M
PLB vs OLZ	Pooled											M
	Tohen2006	361	U	U	L	L	L	L	L	L	H	M
	Vieta 2012	266	U	U	L	L	L	L	L	L	L	L
PLB vs QTP	Pooled											M
	Weisler 2011	808	L	L	L	L	L	H	L	L	H	H
	Young2012	585	U	U	L	L	L	L	L	L	H	M
PLB vs RisLAI	Pooled											H
	Quiroz2010	275	L	L	L	L	L	L	L	L	H	M
	Vieta 2012	267	U	U	L	L	L	L	L	L	H	M
PLB vs PAL	Pooled											M
	Bewaerts 2012	300	L	L	L	L	L	L	L	L	H	M
LIT vs VPA	Pooled											M
	Bowden 2000	278	U	U	L	L	L	L	L	L	H	M
	Calabrese 2005	60	U	U	L	L	L	L	L	L	L	L
	Geddes 2010	220	L	L	H	H	L	L	L	H	L	H
LIT vs CBZ	Pooled											H
	Coxhead 1992	31	U	U	L	L	L	L	L	H	H	H
	Kleindienst 2000	171	L	L	H	H	H	H	L	U	L	H
	Hartong 2003	53	U	U	L	L	L	L	L	L	H	M
LIT vs LTG	Pooled											M
	Bowden 2003	105	U	U	L	L	L	L	L	H	H	H
	Calabrese 2003	292	U	U	L	L	L	L	L	H	H	H
LIT vs IMP	Pooled											H
	Prien 1973a	31	U	U	L	H	L	L	H	H	L	H
	Kane 1982	9	U	U	L	L	L	L	L	L	L	L
	Prien 1984	78	U	U	L	H	L	L	L	L	L	M
LIT vs LIT + IMP	Pooled											H
	Kane 1981	75	U	U	L	L	L	L	H	L	U	M
	Kane 1982	10	U	U	L	L	L	L	L	L	L	L
	Prien 1984	78	U	U	L	H	L	L	L	L	L	M
LIT vs LIT + VPA	Pooled											M
	Geddes 2010	220	L	L	H	H	L	L	L	H	L	H
LIT vs LIT + OXC	Pooled											H
	Vieta 2008	55	L	U	L	L	L	L	L	L	H	M
LIT vs OLZ	Pooled											M
	Tohen2005	431	U	U	L	L	L	L	L	L	H	M
LIT vs QTP	Pooled											M
	Weisler 2011	768	L	L	L	L	L	H	L	L	H	H
VPA vs LIT + VPA	Pooled											H
	Geddes 2010	220	L	L	H	H	L	L	L	H	L	H
VPA vs VPA + ARP	Pooled											H
	Woo 2011	83	U	U	L	L	L	H	L	L	H	H
LTG vs ARP + LTG	Pooled											H
	Carlson 2012	351	U	U	L	L	L	L	L	L	H	M
IMP vs LIT + IMP	Pooled											M
	Kane 1982	11	U	U	L	L	L	L	L	L	L	L
	Prien 1984	72	U	U	L	H	L	L	L	L	L	M
OLZ vs RisLAI	Pooled											M
	Vieta 2012	263	U	U	L	L	L	L	L	L	H	M
	Pooled											M

*L* low risk of bias, *M* moderate risk of bias, *H* high risk of bias, *U* unclear risk of bias

References to studies can be found in the original paper [4]

*ARP* aripiprazole, *CBZ* carbamazepine, *FLX* fluoxetine, *IMP* imipramine, *LIT* lithium, *LTG* lamotrigine, *OLZ* olanzapine, *OXC* oxcarbazepine, *PAL* paliperidone, *PLB* placebo, *QTP* quetiapine, *RisLAI* risperidone long-acting injection, *VPA* valproate

Table 2 represents the contribution matrix of each direct comparison to network estimates. Summating percentage contributions from direct estimates (Table 2) at low, moderate or high RoB according to Table 1, we obtain Table 3, which shows the percentage of contributions from direct comparisons at high, moderate or low risks of bias to each network estimate.

**Table 2:** Contribution matrix for any mood episode relapse (the complete contribution matrix is shown on pp. 84–85 of the Appendix in Miura et al. [4])

	Number of comparisons	PLB vs LIT	PLB vs VPA	PLB vs LTG	PLB vs IMP	PLB vs FLX	PLB LIT+IMP	PLB vs ARP	PLB vs OLZ	PLC vs QTP	PLB RisLAI	PLB vs PAL	LIT vs VPA	LIT vs CBZ	LIT vs LTG	LIT vs IMP	LIT vs FLX	LIT vs LIT+IMP	LIT vs LIT+VPA	LIT vs LIT+OXC	LIT vs OLZ	LIT vs QTP	VPA vs LIT+VPA	VPA vs VPA+ARP	LTG vs VPA+LTG	LTG vs ARP+LTG	IMP vs LIT+IMP	OLZ vs RisLAl
Any mood eplaode																												
PLB vs LIT	10	27.2	5.3	13.7	2.3		0.2	0.0	4.9	5.7	2.1	0.0	3.1	0.0	13.7	1.8		0.7	2.2	0.0	6.9	5.7	2.2	0.0		0.0	0.5	2.1
PLB vs VPA	1	14.2	13.0	7.1	1.2		0.1	0.0	2.5	3.0	1.1	0.0	17.0	0.0	7.1	0.9		0.4	12.2	0.0	3.6	3.0	12.2	0.0		0.0	0.3	1.1
PLB vs LTG	4	9.9	1.9	56.5	0.8		0.1	0.0	1.8	2.0	0.7	0.0	1.1	0.0	17.2	0.6		0.3	0.8	0.0	2.5	2.0	0.8	0.0		0.0	0.2	0.7
PLB vs IMP	2	14.8	2.9	7.4	8.1		0.5	0.0	2.6	3.1	1.1	0.0	1.7	0.0	7.4	23.9		7.9	1.2	0.0	3.7	3.1	1.2	0.0		0.0	8.4	1.1
PLB vs LIT+IMP	1	14.3	2.8	7.1	4.3		1.0	0.0	2.5	3.0	1.1	0.0	1.6	0.0	7.1	10.3		20.5	1.2	0.0	3.6	3.0	1.2	0.0		0.0	14.6	1.1
PLB vs ARP	1	0.0	0.0	0.0	0.0		0.0	100.0	0.0	0.0	0.0	0.0	0.0	0.0	0.0	0.0		0.0	0.0	0.0	0.0	0.0	0.0	0.0		0.0	0.0	0.0
PLB vs. OLZ	2	11.5	2.2	5.8	1.0		0.1	0.0	22.5	2.4	9.5	0.0	1.3	0.0	5.8	0.8		0.3	0.9	0.0	23.0	2.4	0.9	0.0		0.0	0.2	9.5
PLB vs. QTP	2	14.4	2.8	7.2	1.2		0.1	0.0	2.6	23.6	1.1	0.0	1.6	0.0	7.2	0.9		0.4	1.2	0.0	3.7	29.4	1.2	0.0		0.0	0.3	1.1
PLB vs RisLAI	2	4.6	0.9	2.3	0.4		0.0	0.0	9.0	1.0	49.4	0.0	0.5	0.0	2.3	0.3		0.1	0.4	0.0	9.2	1.0	0.4	0.0		0.0	0.1	18.2
PLB vs PAL	1	0.0	0.0	0.0	0.0		0.0	0.0	0.0	0.0	0.0	100.0	0.0	0.0	0.0	0.0		0.0	0.0	0.0	0.0	0.0	0.0	0.0		0.0	0.0	0.0
Manio eplaode																												
PLB vs LIT	7	39.4	11.6	9.5	0.8		0.4	0.0	0.6	5.9	0.2	0.0	9.4	0.0	9.5	0.7		0.5	2.2	0.0	0.8	5.9	2.2	0.0	0.0	0.0	0.1	0.2
PLB vs VPA	1	23.2	16.3	5.6	0.5		0.2	0.0	0.3	3.5	0.1	0.0	21.1	0.0	5.6	0.4		0.3	6.4	0.0	0.5	3.5	6.4	0.0	0.0	0.0	0.1	0.1
PLB vs LTG	3	12.9	3.8	54.3	0.3		0.1	0.0	0.2	1.9	0.1	0.0	3.1	0.0	19.3	0.2		0.2	0.7	0.0	0.3	1.9	0.7	0.0	0.0	0.0	0.0	0.1
PLB vs IMP	1	20.4	6.0	4.9	2.5		0.6	0.0	0.3	3.1	0.1	0.0	4.9	0.0	4.9	23.8		11.0	1.2	0.0	0 4	3.1	1.2	0.0	0.0	0.0	11.6	0.1
PLB vs LIT+IMP	1	21.3	6.0	4.9	1.4		1.1	0.0	0.3	3.0	0.1	0.0	4.8	0.0	4.9	11.3		23.3	1.1	0.0	0.4	3.0	1.1	0.0	0.0	0.0	12.7	0.1
PLB vs ARP	1	0.0	0.0	0.0	0.0		0.0	99.9	0.0	0.0	0.0	0.0	0.0	0.0	0.0	0.0		0.0	0.0	0.0	0.0	0.0	0.0	0.0	0.0	0.0	0.0	0.0
PLB vs OLZ	2	1.2	0.4	0.3	0.0		0.0	0.0	54.7	0.2	20.1	0.0	0.3	0.0	0.3	0.0		0.0	0.1	0.0	2.1	0.2	0.1	0.0	0.0	0.0	0.0	20.1
PLB vs QTP	2	22.2	6.6	5.4	0.4		0.2	0.0	0.3	15.0	0.1	0.0	5.3	0.0	5.4	0.4		0.3	1.3	0.0	0.4	35.3	1.3	0.0	0.0	0.0	0.1	0.1
PLB vs RisLAI	2	0.4	0.1	0.1	0.0		0.0	0.0	19.4	0.1	58.5	0.0	0.1	0.0	0.1	0.0		0.0	0.0	0.0	0.8	0.1	0.0	0.0	0.0	0.0	0.0	20.2
PLB vs PAL	1	0.0	0.0	0.0	0.0		0.0	0.0	0.0	0.0	0.0	100.0	0.0	0.0	0.0	0.0		0.0	0.0	0.0	0.0	0.0	0.0	0.0	0.0	0.0	0.0	0.0
Dapreesive eplaode																												
PLB vs LIT	8	46.5	2.3	8.5	0.8	2.2	0.4	0.0	4.3	6.7	0.5	0.0	1.1	0.0	8.5	0.6	2.2	0.5	1.2	0.0	4.9	6.7	1.2	0.0	0.0	0.0	0.1	0.5
PLB vs VPA	1	21.2	9.1	3.9	0.4	1.0	0.2	0.0	2.0	3.0	0.2	0.0	15.5	0.0	3.9	0.3	1.0	0.2	16.3	0.0	2.2	3.0	16.3	0.0	0.0	0.0	0.1	0.2
PLB vsLTG	3	20.8	1.1	34.3	0.3	1.0	0.2	0.0	1.9	3.0	0.2	0.0	0.5	0.0	2.8.6	0.3	1.0	0.2	0.5	0.0	2..2	3.0	0.5	0.0	0.0	0.0	0.1	0.2
PLB vs IMP	1	22.0	1.1	4.0	7.3	1.1	1.4	0.0	2.1	3.2	0.3	0.0	0.5	0.0	4.0	23.8	1.1	9.9	0.6	0.0	2.3	3.2	0.6	0.0	0.0	0.0	11.3	0.3
PLB vs FLX	2	17.0	0.9	3.1	0.3	39.6	0.1	0.0	1.6	2.4	0.2	0.0	0.4	0.0	3.1	0.2	25.6	0.2	0.4	0.0	1.8	2.4	0.4	0.0	0.0	0.0	0.0	0.2
PLB vs LIT+IMP	1	22.5	1.1	4.1	3.1	1.1	3.2	0.0	2.1	3.2	0.3	0.0	0.6	0.0	4.1	9.1	1.1	25.3	0.6	0.0	2.4	3.2	0.6	0.0	0.0	0.0	12.1	0.3
PLB vs ARP	1	0.0	0.0	0.0	0.0	0.0	0.0	100.0	0.0	0.0	0.0	0.0	0.0	0.0	0.0	0.0	0.0	0.0	0.0	0.0	0.0	0.0	0.0	0.0	0.0	0.0	0.0	0.0
PLB vs OLZ	2	9.8	0.5	1.8	0.2	0.5	0.1	0.0	53.1	1.4	6.6	0.0	0.2	0.0	1.8	0.1	0.5	0.1	0.3	0.0	14.2	1.4	0.3	0.0	0.0	0.0	0.0	6.6
PLB vs QTP	2	12.9	0.7	2.4	0.6	0.6	0.1	0.0	1.2	57.7	0.1	0.0	0.3	0.0	2.4	0.2	0.6	0.1	0.3	0.0	1.4	18.2	0.3	0.0	0.0	0.0	0.0	0.1
PLB vsRisLAI	2	2.8	0.1	0.5	0.0	0.1	0.0	0 0	15.1	0.4	56.3	0.0	0.1	0.0	0.5	0.0	0.1	0.0	0.1	0.0	4.0	0.4	0.1	0.0	0.0	0.0	0.0	19.2
PLB vs PAL	1	0.0	0.0	0.0	0.0	0.0	0.0	0.0	0.0	0.0	0.0	100.0	0.0	0.0	0.0	0.0	0.0	0.0	0.0	0.0	0.0	0.0	0.0	0.0	0.0	0.0	0.0	0.0

*ARP* aripiprazole, *CBZ* carbamazepine, *FLX* fluoxetine, *IMP* imipramine, *LIT* lithium, *LTG* lamotrigine, *OLZ* olanzapine, *OXC* oxcarbazepine, *PAL* paliperidone, *PLB* placebo, *QTP* quetiapine, *RisLAI* risperidone long-acting injection, *VPA* valproate

**Table 3:** Contribution of risks of bias of direct estimates to network estimates

Comparison	Any mood episode relapse		
	Low (%)	Moderate (%)	High (%)
PLB vs LIT	0.2	22.5	77.6
PLB vs VPA	0.1	22.0	77.9
PLB vs LTG	0.1	8.1	91.7
PLB vs IMP	0.5	27.7	71.9
PLB vs LIT + IMP	1.0	46.2	53.1
PLB vs ARP	0.0	100.0	0.0
PLB vs OLZ	0.1	67.2	32.8
PLB vs QTP	0.1	12.0	87.9
PLB vs RisLAI	0.0	86.9	13.2
PLB vs PAL	0.0	100.0	0.0

Contributions of direct comparisons at high, moderate or low risk of bias to mixed or indirect comparisons were calculated as the sum of direct comparisons with corresponding risks of bias, weighted by the contribution matrix

*ARP* aripiprazole, *CBZ* carbamazepine, *FLX* fluoxetine, *IMP* imipramine, *LIT* lithium, *LTG* lamotrigine, *OLZ* olanzapine, *OXC* oxcarbazepine, *PAL* paliperidone, *PLB* placebo, *QTP* quetiapine, *RisLAI* risperidone long-acting injection, *VPA* valproate

**Fig. 2:**
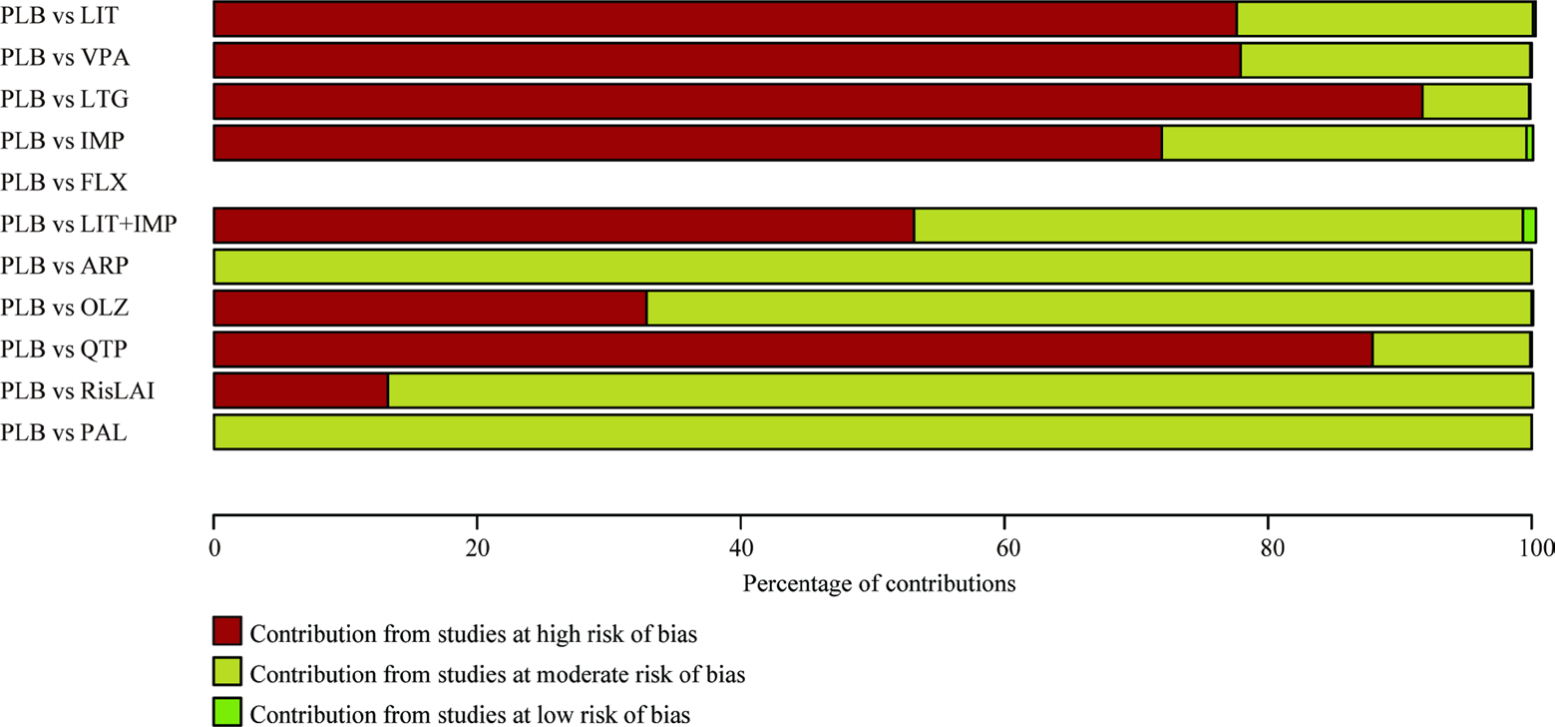
Contributions from studies at high, moderate or low risk of bias to RR to prevent any mood episodes. *ARP* aripiprazole, *CBZ* carbamazepine, *FLX* fluoxetine, *IMP* imipramine, *LIT* lithium, *LTG* lamotrigine, *OLZ* olanzapine, *OXC* oxcarbazepine, *PAL* paliperidone, *PLB* placebo, *QTP* quetiapine, *RisLAI* risperidone long-acting injection, *VPA* valproate (Figure dapted from p. 98 of the Appendix in Miura et al. [4])

For example, 0.2, 22.5 and 77.6 % of the contributions to the network estimate for placebo vs lithium in preventing any mood episode come from direct comparisons with low, moderate and high, respectively, risks of bias. Figure 2 graphically presents the respective contributions for major comparisons in the network.

Thus the network estimate of efficacy of lithium over placebo to prevent any mood episode was based nearly 80 % on studies at high risk of bias and nearly 20 % on studies at moderate risk of bias. This estimate would then be considered quite likely to be biased, either in the direction of under- or over-estimation.

### Contribution of the enrichment design to network estimates

Table 4 shows the percentage of enriched participants for each direct comparison.

**Table 4:** Percentage of enriched participants for each direct comparison

Direct comparisons																											
	PLB vs LIT	PLB vs VPA	PLB vs LTG	PLB vs IMP	PLB vs FLX	PLB LIT+IMP	PLB vs ARP	PLB vs OLZ	PLC vs QTP	PLB RisLAI	PLB vs PAL	LIT vs VPA	LIT vs CBZ	LIT vs LTG	LIT vs IMP	LIT vs LX	LIT vs LIT+IMP	LIT vs LIT+VPA	LIT vs LIT+OXC	LIT vs OLZ	LIT vs QTP	VPA vs LIT+VPA	VPA vs VPA+ARP	LTG vs VPA+LTG	LTG vs ARP+LTG	IMP vs LIT+IMP	OLZ vs RisLAl
Manic (%)	0	0	29	0	–	0	100	58	42	72	100	0	0	26	0	–	22	0	0	0	72	0	100	0	100	41	100
Depression (%)	0	0	79	0	100	0	0	0	58	0	0	0	0	76	0	100	25	0	0	0	28	0	0	100	0	46	0

*ARP* aripiprazole, *CBZ* carbamazepine, *FLX* fluoxetine, *IMP* imipramine, *LIT* lithium, *LTG* lamotrigine, *OLZ* olanzapine, *OXC* oxcarbazepine, *PAL* paliperidone, *PLB* placebo, *QTP* quetiapine, *RisLAI* risperidone long-acting injection, *VPA* valproate

Multiplying Table 4 by the contribution matrix for depressive episode relapse and that for mania episode relapse (Table 2), we obtain Table 5, which shows the percentage of contributions of the enrichment design to network estimates. For example, the NMA estimate of efficacy of placebo versus lithium in preventing depressive episode relapses receives 12.1 % of contributions from studies favouring lithium, 10.5 % from studies favouring placebo, 0.1 % from studies with enrichment design whose direction could not be determined, and the remaining 77.3 % from non-enriched studies.

**Table 5:** Contributions from studies with enrichment design to mixed and indirect estimates

	Depressive episode relapse			Mania episode relapse		
	In favour of the drug to the right (%)	In disfavour of the drug to the right (%)	Enrichment of unknown direction (%)	In favour of the drug to the right (%)	In disfavour of the rug to the right (%)	Enrichment of unknown direction (%)
PLB vs LIT	12.12	10.49	0.05	5.73	6.83	0.24
PLB vs VPA	5.51	0.00	4.82	3.34	0.00	4.18
PLB vs LTG	48.26	0.00	1.94	21.75	0.00	1.51
PLB vs IMP	5.80	5.20	7.43	2.97	4.76	6.03
PLB vs FLX	68.79	0.00	3.02	–	–	–
PLB vs LIT + IMP	17.76	0.00	5.03	13.26	0.00	3.53
PLB vs ARP	0.00	0.00	0.00	99.90	0.00	0.00
PLB vs OLZ	2.59	0.00	2.25	45.83	20.40	0.22
PLB vs QTP	40.87	0.00	2.40	33.53	0.00	1.61
PLB vs RisLAI	0.69	0.00	0.58	72.94	0.00	0.03
PLB vs PAL	0.00	0.00	0.00	100.00	0.00	0.00

When patients were recruited in manic (or depressive) episodes and stabilised with drug A and then after stabilisation randomised to drug A vs drug B, then such patients were considered to have been enriched against manic (or depressive) relapses but not for depressive (or manic) relapses. Contributions of the effects from studies with enrichment design to mixed or indirect comparisons were calculated as the sum of the proportion of such patients in each direct comparison, weighted by the contribution matrix

*ARP* aripiprazole, *CBZ* carbamazepine, *FLX* fluoxetine, *IMP* imipramine, *LIT* lithium, *LTG* lamotrigine, *OLZ* olanzapine, *OXC* oxcarbazepine, *PAL* paliperidone, *PLB* placebo, *QTP* quetiapine, *RisLAI* risperidone long-acting injection, *VPA* valproate

**Fig. 3:**
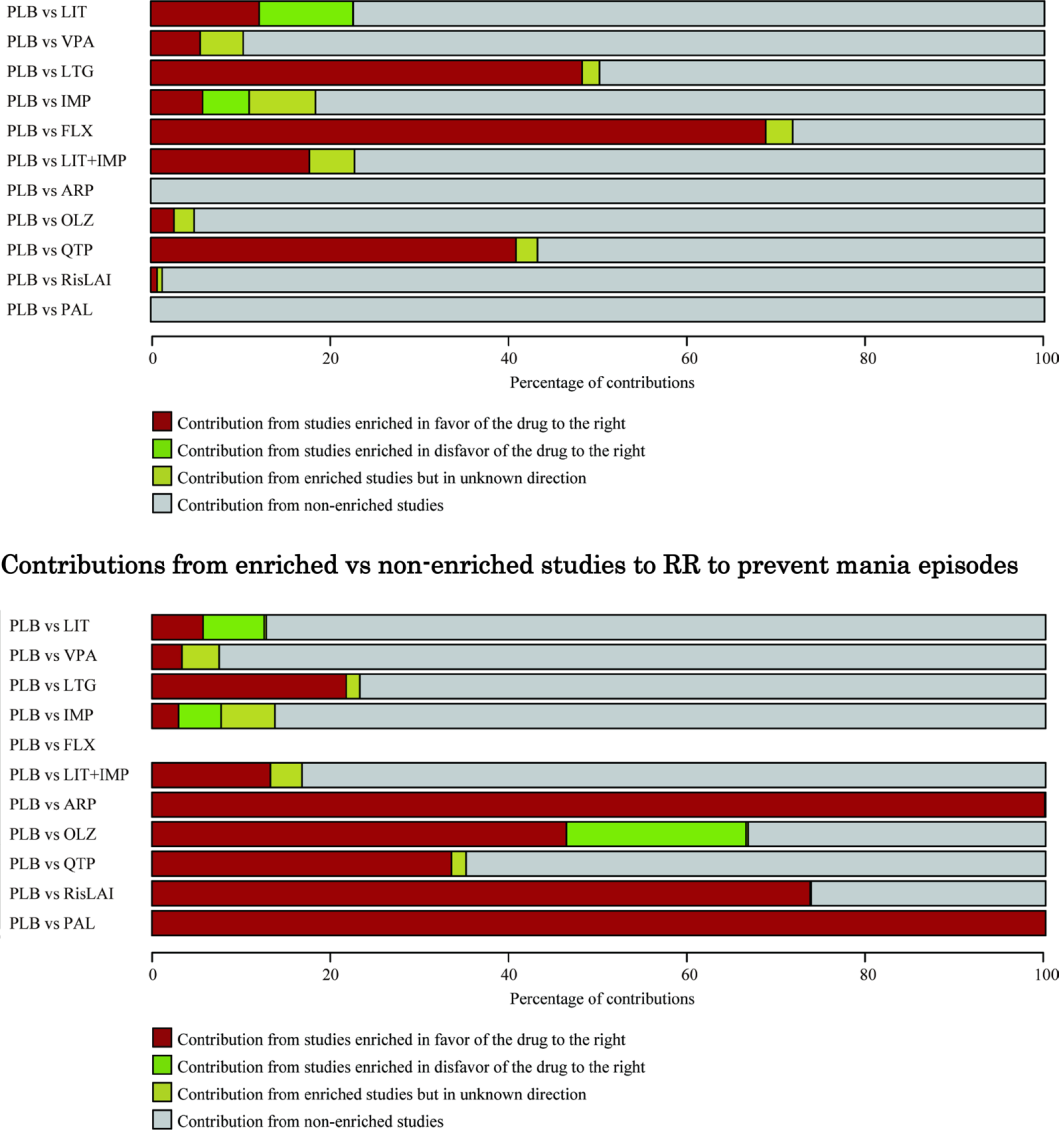
Contributions from enriched vs non-enriched studies to RR to prevent depressive episodes. *ARP* aripiprazole, *CBZ* carbamazepine, *FLX* fluoxetine, *IMP* imipramine, *LIT* lithium, *LTG* lamotrigine, *OLZ* olanzapine, *OXC* oxcarbazepine, *PAL* paliperidone, *PLB* placebo, *QTP* quetiapine, *RisLAI* risperidone long-acting injection, *VPA* valproate (Figures adapted from p. 90 of the Appendix in Miura et al. [4])

We graphically showed the percentages of contributions of enriched vs non-enriched studies to effect estimates of main comparisons against placebo in the network (Fig. 3).

Thus, the network estimate of the efficacy of lithium vs placebo to prevent a depressive episode received a small contribution from studies enriched in favour of lithium, and a similarly small contribution from studies enriched in favour of placebo but the bulk of the evidence was from non-enriched studies. By contrast, the network estimates of fluoxetine or lamotrigine in the prevention of depressive episodes received nearly half or more contribution from studies enriched in favour of the active drugs: it is quite possible that the network estimates for these drugs are overestimated.

## Discussion

We have demonstrated how to appraise the impact of study limitations of included studies on each estimate obtained in the NMA according to the GRADE system in a transparent and quantitative manner, first in the case of standard risks of bias as assessed with the Cochrane method and then also in the case of study limitations which have clear directions and have complex repercussion in the network.

The GRADE framework has been developed to provide a common, sensible method to assess quality of evidence and the strength of recommendation, and successfully applied to conventional pair-wise meta-analyses and clinical guidelines. However, it is difficult to apply the GRADE to NMAs mainly because of the complexity of NMAs. For, in NMA, risk of bias for mixed or network estimates are hard to evaluate, especially in a large network, because mixed estimates are calculated from both direct and indirect estimates with different contributions.

With *netweight*, a command for NMA in STATA [8], we can obtain the contribution matrix showing contributions from each direct comparison to the network estimates even in a large network like our example, and then we can calculate the composition of each level of risk of bias in network estimates quantitatively. We have demonstrated and exemplified that this method, first presented by Salanti et al. [3], is flexible enough to accommodate other sources of bias, including even those which have directions such as the enrichment design in our case.

Admittedly assessments of RoB and GRADE contain strong elements of judgment. Our endeavors represent quantification of these judgments in a reasonable and explicit way and represents important advance in making these judgments more transparent to consumers of evidence (patients, clinicians and policy makers). However we must remember that in essence they are attempts at quantification of in part qualitative statements.

One important consideration when downgrading for study limitation is whether actually studies at high risk of bias give materially different results from those at low risk of bias. If the disagreement is significant, researcher might choose to base their conclusions only on studies at low risk of bias. When both sources of evidence are in agreement, some reviewers might be reluctant to downgrade for study limitations. When disagreement is not substantial and yet not negligible, as it is the case in our example, appropriate statistical methodology should be applied to quantify the potential impact of those high risk of bias studies. In order to examine if studies rated at high RoB do in fact differ or not differ in effect estimates from those rated at low RoB, one solution might be to run subgroup NMA (or meta-regression) to compare the results among those with high RoB and those with low RoB. Others may argue however that, comparing two scenarios where, in one case, all high quality studies provide similar results and, in another case, half are high quality and half are low quality yet both provide similar results, the rating for the resultant meta-analytic results should nonetheless be higher for the former than for the latter. In practice, few network meta-analyses would have enough power to detect material differences between high and low risk of bias studies, so that the question about comparability of results between low and high risk of bias studies has to be answered by large-scale empirical studies [11, 12]. These studies have provided evidence that some risk of bias components might be important when the outcome is not mortality.

*Netweight* can calculate contributions of each direct comparison to the entire network, and therefore the ranking of treatments. The present paper focused on the evaluation of the confidence placed on pairwise treatment effects estimated via NMA rather than treatment ranking. Although the reporting of treatments’ ranking has become increasingly popular and can be clinically useful, it is only an auxiliary output and researchers are warned against consideration of the ranking in isolation from the effect sizes. We therefore think that it is clinically more meaningful and important to evaluate the pairwise effect sizes rather than globally assess the quality of the network evidence as a whole.

In future attempts to apply the GRADE system to NMAs, a systematic and quantitative approach to evaluating how study limitations of individual studies contribute to each network estimate is recommended and should also be endorsed by scientific journals across the field of evidence synthesis.

## Footnotes

1 The STATA command will be in the form of netweight effect_size SE_of_effect_size treatment1 treatment2   where each row in the dataset represents the effect size and its standard error for a study comparing treatment1 and treatment2. For more details, please see [8] and [10].
